# The effect of genistein on cisplatin induced ototoxicity and oxidative stress

**DOI:** 10.1016/j.bjorl.2021.07.001

**Published:** 2021-07-30

**Authors:** Mehmet Tan, Yüksel Toplu, Emrah Varan, Emrah Sapmaz, Onural Özhan, Hakan Parlakpınar, Alaadin Polat

**Affiliations:** a Inonu University, Medical Faculty, Department of Otorhinolaryngology, Malatya, Turkey; b Gaziosmanpaşa University, Medical Faculty, Department of Otorhinolaryngology, Tokat, Turkey; c Inonu University, Medical Faculty, Department of Medical Pharmacology, Malatya, Turkey; d Inonu University, Medical Faculty, Physiology Department, Malatya, Turkey

**Keywords:** Genistein, Ototoxicity, Cisplatin, Oxidative stress

## Abstract

**Objective:**

Cisplatin is an antineoplastic agent used in adults and children for the treatment of various malignant diseases. It can cause irreversible ototoxicity. Genistein is a phytoestrogen. Genistein functions as an antioxidant and cell cycle inhibitor by inhibiting the DNA topoisomerase and tyrosine protein kinase enzymes. The protective effect of genistein in preventing cisplatin-induced ototoxicity and levels of the oxidative stress was investigated.

**Methods:**

32 Sprague Dawley rats were used in 4 groups (control, cisplatin, cisplatin + genistein, genistein). Otoacoustic emission measurements of the distortion product were performed on the 1st, 2nd and 5th days of the test protocol. Serum malondialdehyde, superoxide dismutase, catalase, glutathione peroxidase, total antioxidant status, total oxidant status and oxidative stress index measurements were made.

**Results:**

The hearing of the cisplatin + genistein group was found to be better than that of the cisplatin group. While the malondialdehyde, total oxidant status and oxidative stress index parameters decreased significantly in the cisplatin + genistein group compared to the cisplatin group, superoxide dismutase increased significantly (*p* < 0.05).

**Conclusion:**

Genistein showed positive effects against ototoxicity with its antioxidant effect.

**Level of evidence:**

Level 3.

## Highlights

• Cisplatin is an antineoplastic agent used malignant diseases.
• Cisplatin ototoxicity is generally bilateral, irreversible, and progressive.
• Genistein is a phytoestrogen.
• Genistein functions as antioxidant and cell cycle inhibitor by inhibiting DNA topoisomerase.
• Genistein showed positive effects on ototoxicity with its antioxidant.

## Introduction

Ototoxicity is cellular degeneration and dysfunction in the cochlea and vestibular organ caused by various drugs and chemicals. Cisplatin is an antineoplastic agent used in adults and children for the treatment of various malignant diseases such as head and neck tumors and urogenital system, central nervous system, respiratory system, and esophageal cancers.^1^ Besides nephrotoxicity and irreversible ototoxicity, neurotoxicity, gastrointestinal tract and bone marrow toxicity are among the significant dose-limiting side effects of cisplatin.^2^ It is commonly used in children, as well as adults.^3^ Cisplatin-induced hearing (CIO) loss was first defined in 1972.^4^ Cisplatin ototoxicity is generally bilateral, irreversible and progressive, and it involves high frequencies and starts within days or weeks. Ototoxicity is dose-dependent and cumulative, and as the dose increases, its effects also increase.^5^, ^6^ The causation of CIO is not clear. The most significant mechanisms are an increase in the generation of reactive oxygen species (ROS) and a reduction in the antioxidant enzyme system in the cochlea.^7^ Disruption of this balance within the cell is likely to result in cochlear cell damage and death. This balance is aimed to be restored by using antioxidant agents. In the relevant literature, various agents have been reported to be used in restoring this balance and reducing ROS formation.^8^

The compound genistein is an important nutraceutical molecule that is abundantly found in soybeans. Genistein (GST) is a phytoestrogen that can display a wide range of pharmacological effects, especially tyrosine kinase inhibition, in animal cells. Genistein is the focus of epidemiological studies and various studies conducted with experimental animals due to its potential of beneficial effects on health with daily dietary intake. It is reported that more than 3600 studies have been conducted to examine the biological effectiveness of genistein in the last 10 years. However, more studies should be carried out to examine the beneficial effects of genistein based on the current data in the literature.^9^ Genistein functions as an antioxidant and cell cycle inhibitor by inhibiting the DNA topoisomerase and tyrosine protein kinase enzymes.^10^

There is no study that examines the effect of GST on CIO in the literature. This study assessed ototoxic effects using distortion product otoacoustic emission (DPOAE) measurements. The study also evaluated the serum levels of the oxidative stress parameter malondialdehyde (MDA), total oxidant status (TOS) and oxidative stress index (OSI), as well as antioxidant contents superoxide dismutase (SOD), catalase (CAT), glutathione peroxidase (GPX), reduced GSH, and total qntioxidant status (TAS). The primary objective of this study was to evaluate the otoprotective effect of GST in CIO in rats; the second aim is to compare serum oxidant and antioxidant parameters in the GST group, CIS group, GST + CIS group, and control group.

## Materıal and methods

Ethics committee approval was obtained from the Experimental Animals Ethics Committee with the protocol number of 2019/A-45. The study used 32 female Sprague-Dawley rats. Three months old rats with body weights between 200 g and 300 g and with no hearing problems in the day 1 emission measurements were included in the experiment. Since the comparisons between the four groups in terms of the biochemical variables predicted an effect size of 0.7434, the minimum sample size per group was calculated as 8 (n = 8) for 90% power and a 95% Confidence Interval. The experimental animals were kept in the laboratory for 12 h under light and 12 h in the dark under conditions automatically regulated at an ambient temperature of 22 ± 1 °C and 45%–50% humidity. The animals were given standard pellets and fresh tap water every day.

Group 1 (Control) – The group (n = 8) which was given vehicle solvent of genistein DMSO: EtOH (2:1) 300 µL intraperitoneally once a day for five days one hour before the IP injection of vehicle solvent of cisplatin injection water in day 1.

Group 2 (CIS) – The group (n = 8) which was given vehicle solvent of genistein DMSO: EtOH (2:1) 300 µL intraperitoneally once a day for five days one hour before 4 doses of 16 mg/kg CIS IP injections in 6-h intervals in day 1.

Group 3 (CIS-GST) – The group (n = 8) which was given 10 mg/kg genistein intraperitoneally once a day for five days one hour before 4 doses of 16 mg/kg CIS IP injections in 6-h intervals in day 1.

Group 4 (GST) – The group (n = 8) which was given 10 mg/kg genistein intraperitoneally once a day for five days one hour before the IP injection of vehicle solvent of cisplatin injection water in day 1.

The DPOAE measurements of the rats under ketamine/xylazine (100 mg kg/10 mg kg) anesthesia were performed at the Experimental Animals Production and Research Center on the 1st, 2nd and 5th days of the experimental protocol. The DP-gram and input/output functions between 0.9961 and 8.0003 Hz in the right ears of the rats were recorded. The measurements were made according to the methods we previously performed successfully in studies published in 2014 and 2016. The measurements were made using a GSI Audera DPOAE device (Grason Stadler, Madison, WI, USA). The device was calibrated before every measurement using the automated measurement system. The measurements were made in a quiet cabin isolated from surrounding sounds. The primary stimulus levels were equalized at 65 dB (L1 = L2). To get the strongest responses, two different frequencies (f1 and f2) were prepared at f2: f1 = 1.22. The DPOAE measurements were made on day 0, 1 and 5 at the frequencies of 996, 1,266, 1,582, 2,004, 2,519, 3,176, 3,996, 5,039, 6,351, 8,004 and 10,078 Hz. We aimed to evaluate the oxidative stress level and antioxidant effect that play a role in the pathophysiology of CIO. Approximately 5 mL of intracardiac blood was collected after the emission measurements on the 5th day of the experiment. MDA, SOD, CAT, GPX, TAS, TOS and OSI levels were determined spectrophotometrically by PCR.

### Biochemical measurements

SOD activity was calculated according to the method described by Sun et al.^11^ According to this method, SOD activity is observed by reducing the superoxide-Nitro Blue Tetrazolium (NBT) produced by the xanthine/xanthine oxidase effect. 1 U SOD is an enzyme activity that inhibits NBT reduction by 50%, and the results are given as U/mg protein.

Catalase activity was calculated according to Aebi’s method.^12^ This method is based on the principle of measuring the absorbance reduction in the (UV) spectrophotometer as a result of resolving H_2_O_2_ which is an active chemical substance into water and oxygen in the presence of the CAT enzyme. This reduction in the absorbance is directly proportionate to the CAT enzyme activity. In UV spectrophotometry analysis, H_2_O_2_ provides the maximum absorbance at the wavelength of 240 nm.

An enzymatic reaction was initiated by adding H_2_O_2_ to determine GPX activity according to the method suggested by Paglia and Valentine.^13^ Tube GSH consisted of sodium azide NADPH and glutathione reductase. Changes in 340 nm absorbance were observed using a spectrophotometer. The results are presented as µ/mg protein. The absorbance values of the GSH content were extrapolated with the aforementioned method. The result was derived from the glutathione standard curve and is presented as GSH (Umol/L).

MDA, which a lipid peroxidation product, was determined according to the method reported by Uchiyama et al.^14^ The supernatant of this pink colored compound was extracted in the *N*-butanol phase by reacting MDA with Thiobarbituric Acid (TBA). This method is based on measuring the formed supernatant by a spectrophotometer at 535 and 520 nm. The standards in various concentrations were prepared with 1,1,3,3 tetramethoxypropane, and the results were evaluated with the standard plot drawn from them and are presented as µmoL/L.

Serum TAC levels were measured using the automated measurement method. The results are presented as mmol Trolox Equivalent/L unit.^15^

Total Oxidant Status (TOS) measurement was made using an immunostimulant plate reader with the BioTek HT Synergy Gen 5 software and a TOS kit set (Rel Assay Diagnostics, Turkey).^16^ Ferric ions in an acidic environment form a colored complex with xylenol orange. The color density, which can be measured spectrophotometrically, is proportional to the total quantity of the oxidant molecules in the sample. The experiment was calibrated with hydrogen peroxide, and the results are presented in micromolar hydrogen peroxide equivalent per liter (μmoL H_2_O_2_ equivalent/L). OSI levels were calculated as TOS/TAC and are presented as arbitrary units.^17^

### Statistical analyses

#### Statistical analyses of otoacoustic measurements

IBM SPSS Statistics ver. 21.0 (IBM Co., Armonk, NY, USA) was used for the statistical analyses. Kolmogorov-Smirnov test was used to determine whether the groups displayed normal distribution. The groups were compared using Kruskal–Wallis analysis of variance and Mann–Whitney *U* tests when the DPOAE measurements displayed a non-normal distribution. Wilcoxon paired-samples tests were used in the analysis of the dependent variables.

#### Biochemical statistical analyses

The statistical analyses were conducted with a computer package program. One-way analysis of variance (ANOVA) was used to compare the groups which displayed normal distribution and were expressed as mean ± standard deviation, while Tukey’s test was used for the groups that showed homogeneous variance in the multiple comparisons. Kruskal Wallis H analysis was used to compare the groups which did not show a normal distribution in terms of MDA and TOS, which are expressed as median (minimum–maximum), while Mann–Whitney *U* test was used in the multiple comparisons. The statistical significance level was accepted as *p* < 0.05.

## Results

### Otoacoustic measurements

**Figure 1:**
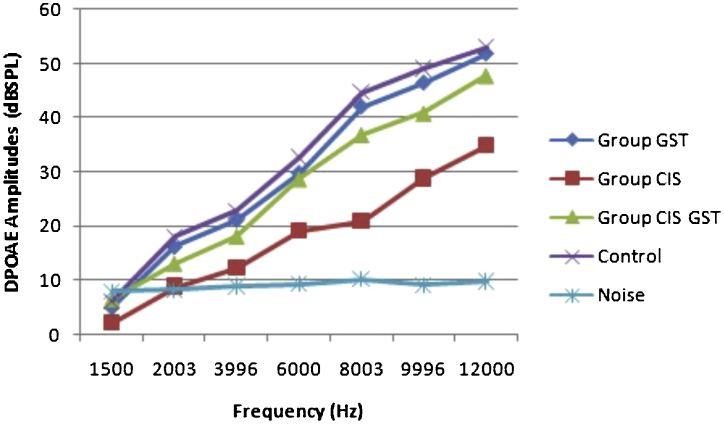
Distortion product otoacoustic emission (DPOAE) amplitudes measured on day 1. CIS, cisplatin; GST, genistein; CIS + GST, genistein plus cisplatin; SPL, sound pressure level.

**Figure 2:**
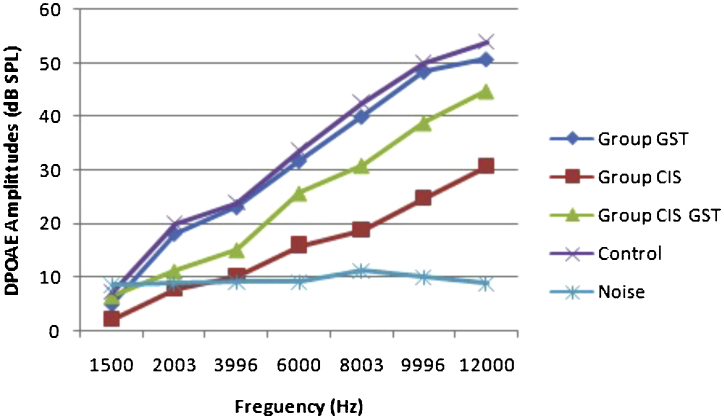
Distortion product otoacoustic emission (DPOAE) amplitudes measured on day 2. CIS, cisplatin; GST, genistein; CIS + GST, genistein plus cisplatin; SPL, sound pressure level.

**Figure 3:**
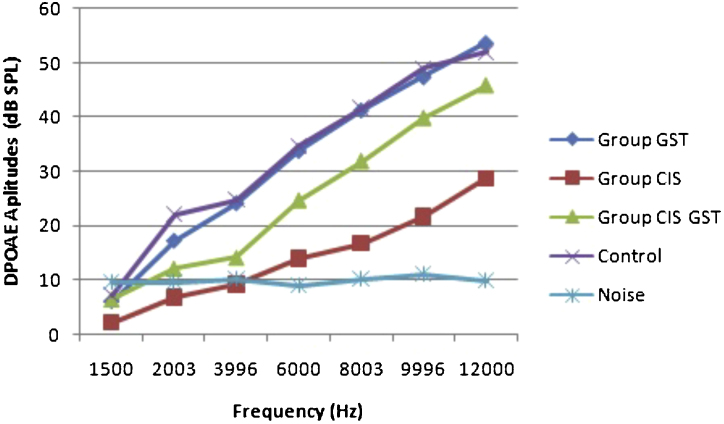
Distortion product otoacoustic emission (DPOAE) amplitudes measured on day 5. CIS, cisplatin; GST, genistein; CIS + GST, genistein plus cisplatin; SPL, sound pressure level.

**Figure 4:**
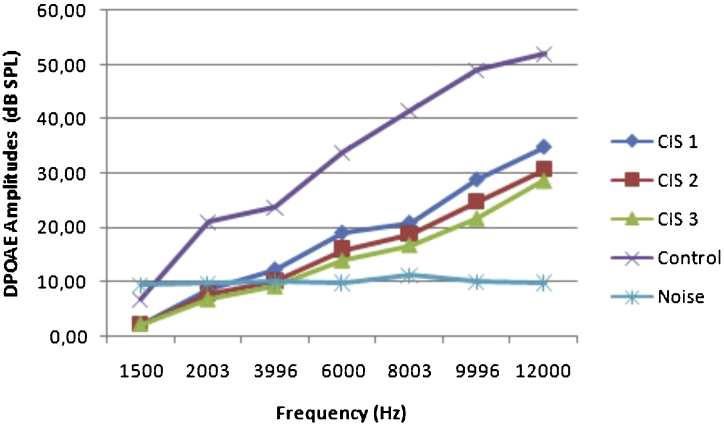
Comparison of the control group and CIS group distortion product otoacoustic emission (DPOAE) measurements on the 1st, 2nd and 5th days.

**Figure 5:**
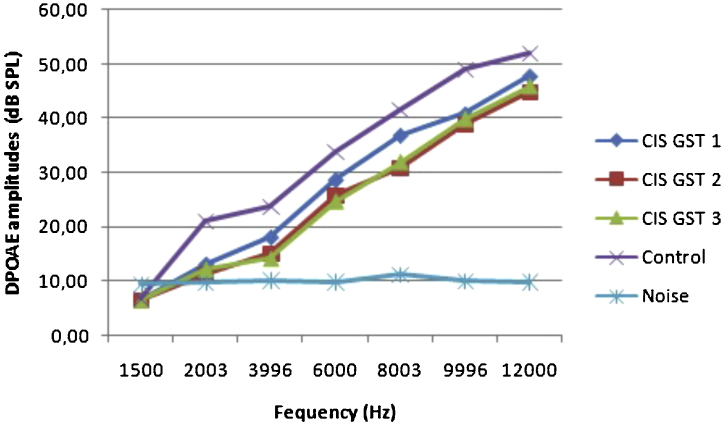
Comparison of the control group and CIS-GST group distortion product otoacoustic emission (DPOAE) measurements on the 1st, 2nd and 5th days.

The before and after drug plots of all groups are presented in Figure 1, Figure 2, Figure 3, Figure 4, Figure 5. No significant differences were found among the GST, CIS-GST and control groups in terms of the DPOAE values in the analysis conducted on the 1st day after the administration of the drugs (*p* > 0.05). However, there was a significant difference in the CIS group (*p* < 0.05) (Fig. 1). In the hearing tests carried out on the 2nd day, the CIS and CIS-GST groups had significant hearing loss in comparison to the control group (*p* < 0.05) (Fig. 2). There was no statistically significant difference between the GST and control groups (*p* > 0.05). In the comparison of the CIS group to the CIS-GST group, the CIS-GST group had better hearing levels. There were statistically significant differences between the DPOAE values of the groups especially at frequencies higher than 6000 Hz (*p* < 0.05). In the analysis conducted on the 5th day, the CIS group had significantly lower hearing levels (*p* < 0.05) (Fig. 3). There were no statistically significant differences between the CIS-GST and CIS groups at 1500, 2003 and 3996 Hz (*p* > 0.05). The hearing values of the rats in the CIS group decreased on the 2nd and 5th days; however, this decrease was not statistically significant (*p* > 0.05) (Fig. 4). The hearing values in the CIS-GST group decreased in the measurements made on the 2nd and 5th days in comparison to the measurement made on the 1st day (*p* > 0.05); however, there was no significant difference between the measurements made on the 2nd and 5th days (*p* > 0.05) (Fig. 5).

### Biochemical analyses

Table 1 shows the scores of the antioxidant parameters studied from the serums of the samples after the blood samples taken at the end of the experiment were centrifuged. The TOS and OSI levels were significantly higher in the CIS group than the control group, while the cisplatin and GST supplement significantly reduced all these toxicity parameters. Additionally, the serum SOD, CAT, GPX and TAC contents significantly decreased in the CIS group, while the GST treatment significantly increased all parameter levels.

**Table 1:** Comparison of serum oxidative stress parameters and antioxidant status.

Group	MDA	SOD	CAT	GPx	TOS	TAS	OSI
	µmoL/L	U/mL	K/L	U/mL	µmoL H_2_O_2_ Eq/L	µmoL Trolox Eq/L	Arbitrary unit
Control	3.23 (2.76–4.92)	0.61 ± 0.09	4.39 ± 1.62	4.68 ± 0.96	21.11 (13.53–30.47)	1.11 ± 0.20	20.70 ± 7.86
CIS	4.08^a^ (2.33–6.16)	0.36 ± 0.08^a^	2.47 ± 0.76^a^	2.25 ± 0.81^a^	36.86^a^ (26.16–50.24)	0.75 ± 0.26^a^	53.06 ± 14.65^a^
CIS GST	3.40^b^ (2.50–4.50)	0.46 ± 0.10^b^	2.68 ± 1.32^b^	2.94 ± 0.90^b^	31.96^b^ (18.28–37.90)	0.97 ± 0.18^b^	33.14 ± 9.55^b^
GST	1.57^a^ (1.15–2.50)	0.50 ± 0.09^a^	3.54 ± 1.44^a^	3.11 ± 1.03^a^	23.34^a^ (19.77–39.84)	0.96 ± 0.16^a^	27.64 ± 7.18^a^

MDA, serum levels of the oxidative stress parameter malondialdehyde; TOS, total oxidant status; OSI, oxidative stress index; SOD, antioxidant contents superoxide dismutase; CAT, catalase; GPX, glutathione peroxidase; TAS, total antioxidant status.

a *p* < 0.05 versus control group.

b *p* < 0.05 versus cisplatin group.

## Discussion

Various reasons have been suggested in the pathogenesis of drug induced ototoxicity. Previous studies have found that cisplatin caused the formation of ROS which initiate inflammatory reaction and oxidative events by accumulating in the cell or being in the forefront. Considering the molecular mechanisms of CIO, researchers observed an increase in reactive oxygen radicals, reduction in the antioxidant system of the inner ear (glutathione peroxidase, glutathione reductase, catalase) and apoptosis with the activation of the caspase system.^18^, ^19^ Cisplatin activates NOX-3 (nicotinamide adenine dinucleotide phosphate Oxidase 3 isoform). This enzyme increases the production of the superoxide radical by increasing the production of hydrogen peroxide. Hydrogen peroxide is catalyzed by iron, and the free radicals that are formed react with fatty acids in the cell membrane, thus leading to cell damage and death.^20^ In vitro studies on CIO showed that the outer hair cells in the basal part are affected first, and then, CIO proceeds in the apical direction. It then affects the inner hair cells, spiral ganglion, and stria vascular. Cisplatin ototoxicity is generally bilateral, irreversible, and progressive, and it involves high frequencies and starts within days or weeks. Hearing loss tends to progress over time.^21^, ^22^, ^23^ It is observed in 25%–50% of patients who receive tinnitus cisplatin treatment, and it continues for a year in 38% of them.^24^

This study used noninvasive and objective DPOAE in determining cochlear hearing loss. DPOAE reveals the effect of cisplatin on hearing before the occurrence of changes in pure tone audiometry. The most significant characteristic of DPOAE is the diagnosis of the early stages of sound processing and evaluation of the biomechanical activity of the outer hair cells.^25^, ^26^ This study found a statistically significant decrease at all frequencies in the cisplatin group with DPOAE. This significant decrease in the cisplatin group was due to CIO. In the comparison of the hearing tests carried out on the 2nd day in the CIS group to the CIS-GST group, the CIS-GST group had better hearing levels. There were statistically significant differences between the DPOAE values especially at frequencies higher than 6000 Hz (*p* < 0.05). In the evaluation made on the 5th day, the CIS group had significantly lower hearing levels (*p* < 0.05). There were no statistically significant differences between the CIS-GST and CIS groups at 1500, 2003 and 3996 Hz (*p* > 0.05).

Studies showed that genistein, a soy phytochemical, is an antioxidant, an inflammatory modulator, and an inhibitor of epigenetic changes in preclinical models.^27^ Studies have still been conducted on the free radical repellant (antioxidant), anti-thrombotic, antihypertensive, antiallergic, anti-inflammatory, antiapoptotic, and recently, anticancerogenic activities of flavonoids. Various studies have examined the antioxidant effect mechanisms of flavonoids and reported different activities. Flavonoids can bind metals as metal:flavonoid at various ratios. The antioxidant properties of some flavonoids may emerge with chelation of metals, especially iron and copper. The movements of metal ions are critical cofactors in the Fenton reaction. Thus, their chelation with flavonoids makes metals unusable for this type of reactions.^28^ Flavonoids are asserted to induce xanthine oxidase inhibition. Inhibition of xanthine oxidase may help prevent superoxide formation.^29^ The study also evaluated the present hearing results and serum biochemical parameters including new oxidant and antioxidant contents such as TOS, TAC and OSI measured in the blood. SOD, CAT, GPX and GSH caused a decrease in the level of TAS antioxidant enzymes, and their contents increased the level of MDA, TOS and OSI. GST caused a change in the molecular state that produced all this ototoxic effect (Table 1). The cause of the cochlear damage and ototoxicity might have increased lipid peroxidation and oxidative stress parameters and reduced the activity of antioxidant systems. Additionally, more studies should be conducted to assess the role of GST at different dosages in prevention and treatment of CIO.

## Conclusion

In conclusion, our findings show for the first time in the available literature that genistein may protect against cisplatin ototoxicity by raising the levels of antioxidant enzymes and lowering the levels of oxidant parameters. We suggest that genistein may be a viable option against cisplatin ototoxicity.

## Funding

The research was supported by the Research Fund of TheInonu University, Malatya, Turkey.

## Conflicts of interest

The authors declare no conflicts of interest.
